# Ibrutinib protects T cells in patients with CLL from proliferation-induced senescence

**DOI:** 10.1186/s12967-021-03136-2

**Published:** 2021-11-22

**Authors:** Joanne E. Davis, Chia Sharpe, Kylie Mason, Constantine S. Tam, Rachel M. Koldej, David S. Ritchie

**Affiliations:** 1 ACRF Translational Research Laboratory, Royal Melbourne Hospital, Melbourne, Australia; 2 Faculty of Medicine, Dentistry and Health Sciences, University of Melbourne, Melbourne, Australia; 3 Clinical Haematology, Peter MacCallum Cancer Centre and Royal Melbourne Hospital, Melbourne, Australia

**Keywords:** Chronic Lymphocytic Leukaemia, Bruton’s tyrosine kinase inhibitors, T cells, Proliferation, Senescence

## Abstract

**Background:**

The development of Bruton’s tyrosine kinase inhibitors (BTKi) for the treatment of chronic lymphocytic leukaemia (CLL) has provided a highly effective and relatively non-toxic alternative to conventional chemotherapy. Some studies have shown that BTKi can also lead to improvements in T cell immunity in patients despite in vitro analyses suggesting an immunosuppressive effect of BTKi on T cell function.

**Methods:**

In this study, we examined both the in vitro effect and long-term in vivo effect of two clinically available BTKi, ibrutinib and zanubrutinib. Additional in vitro assessments were undertaken for a third BTKi, acalabrutinib. Immune subset phenotyping, cytokine secretion, T cell degranulation and proliferation assays were performed on peripheral blood mononuclear cells isolated from untreated CLL patients, and CLL patients on long-term (> 12 months) BTKi treatment.

**Results:**

Similar to prior studies we observed that long-term BTKi treatment normalises lymphocyte subset frequency and reduces PD-1 expression on T cells. We also observed that T cells from patients taken prior to BTKi therapy showed an abnormal hyper-proliferation pattern typical of senescent T cells, which was normalised by long-term BTKi treatment. Furthermore, BTKi therapy resulted in reduced expression of the T cell exhaustion markers PD-1, TIM3 and LAG3 in late generations of T cells undergoing proliferation.

**Conclusions:**

Collectively, these findings indicate that there are critical differences between the in vitro effects of BTKi on T cell function and the effects derived from long-term BTKi exposure in vivo. Overall long-term exposure to BTKi, and particularly ibrutinib, resulted in improved T cell fitness in part due to suppressing the abnormal hyper-proliferation of CLL T cells and the associated development of T cell senescence.

**Supplementary Information:**

The online version contains supplementary material available at 10.1186/s12967-021-03136-2.

## Introduction

Chronic lymphocytic leukaemia (CLL) is the most common adult leukaemia in the western world and is associated with significant perturbations of cellular and humoral immunity including an excess of terminally differentiated T cells and elevated expression of exhaustion markers including Program cell Death molecule-1 (PD-1) and Cytotoxic T Lymphocyte Antigen-4 (CTLA-4) (Reviewed in [1]). Compounding these CLL-specific immune defects are the additional immunosuppressive effects of prior lympholytic conventional chemotherapy, homeostatic proliferation of dysfunctional post-chemotherapy T cell clones [2] and the underlying effects of aging-related T cell senescence (Reviewed in [3]). More recently, Bruton’s tyrosine kinase (BTK) inhibitors, particularly the first generation BTK inhibitor (BTKi) ibrutinib, have become a mainstay CLL therapy. Initially indicated for the treatment of relapsed CLL, [4] more recently BTKi has been incorporated as part of frontline therapy [5] and thereby represent an opportunity for chemotherapy-free treatment of CLL, potentially reducing the impact of lymphodepleting chemotherapy and promoting long-term preservation of immunity. In addition, ibrutinib also inhibits other Tec family kinases, including IL-2-inducible T cell kinase (ITK) in T cells, leading to enhanced T_H_1 immune responses [6]. The second generation more selective BTKi, including zanubrutinib and acalabrutinib, have similar clinical efficacy in control of CLL [7–9] but with limited ITK inhibition and therefore potentially a decreased impact on T cell function.

Although there is substantial evidence of the negative impact of ibrutinib-induced suppression of NK cell function [10], including inhibition of ADCC [11], the direct impact of ibrutinib or other BTKi on the in vitro function of T cells has been less well described. However, ibrutinib is known to inhibit the activation, degranulation and proliferation of healthy donor T cells [12]. In a mouse model of CLL ibrutinib treatment resulted in significantly lower numbers of CD8 + effector T-cells, with lower expression of activation markers, as well as impaired proliferation and effector function. However, co-administration of ibrutinib was associated with increased efficacy of checkpoint blockade therapy and improved CD8^+^ T-cell effector function and control of CLL suggesting that modulation of T cell function by ibrutinib may lead to enhanced T cell function in vivo [13]. Clinically, early observations in ibrutinib-treated patients suggested an immunosuppressive effect with increased opportunistic infections in heavily pre-treated patients with CLL who subsequently were treated with ibrutinib [14–17]. These findings have been linked to the impairment of macrophage function by ibrutinib [18, 19] in addition to cumulative impairment of cellular immunodeficiency induced by prior therapies and/or persistent immune dysfunction exerted by the effects of the CLL microenvironment. Importantly, there appear to be few opportunistic infections recorded in previously untreated patients with CLL who receive ibrutinib as their first therapy [20]. Indeed, frontline ibrutinib therapy has been associated with a broadening of T cell repertoire and a lower rate of opportunistic infections [21, 22]. Furthermore, others have shown that ibrutinib therapy, but not therapy with the more selective second generation BTKi acalabrutinib, over the course of 6 cycles of treatment is associated with improved CD4^+^ and CD8^+^ T cell numbers, possibly by preventing ITK-dependent activation-induced T cell death [23]. This study also showed a differential BTKi effect of ibrutinib-induced reduction of expression of the T cell suppression surface markers PD-1 and CTLA-4 and overall suggested that ibrutinib exposure improves the number and functional status of T cell populations. More recently it has been reported that long-term treatment with ibrutinib significantly restored T-cell proliferative ability, degranulation, and cytokine secretion in peripheral blood samples from patients with CLL [24]. We have also demonstrated that long-term exposure to ibrutinib in vivo may return the T cell function of patients with advanced mantle cell lymphoma (MCL) to that of healthy donors [25]. Overall, this improved T cell fitness following ibrutinib therapy may in turn be able to be exploited to improve the generation and efficacy of subsequent adoptive CAR-T cell therapy in CLL [26] or other B cell malignancies including MCL [27].

In this study we wished to explore the impact of first and second generation clinically available BTKi (ibrutinib, acalabutinib and zanubrutinib) on T cell function in vitro and assess whether those effects translated to changes in post-proliferation T cell exhaustion following long-term exposure to BTKi in patients with CLL.

## Methods

### Patient cohort

Blood samples were collected from CLL patients whose disease was refractory to previous treatment and were about to commence either single-agent ibrutinib or zanubrutinib therapy. CLL patients were treated with ibrutinib under compassionate access at the Peter MacCallum Cancer Centre, Melbourne, Australia or zanubrutinib under a Phase I clinical trial (NCT02343120 [9]). Baseline samples were collected prior to treatment with the specified BTKi and long-term treatment samples were collected after 12 months on zanubrutinib therapy (n = 8), and between 12–24 months on ibrutinib therapy (n = 7) (Additional file 2: Table S1). Age-matched Healthy Donor (n = 7) samples were used in ex vivo analysis comparing baseline with long-term treatment samples from CLL patients. In vitro analysis examining the effects of short-term BTKi treatment on T cell function used samples from a separate cohort of Healthy Donor (n = 6), and treatment-naive CLL patients (n = 11).

### Patient sample processing

Peripheral blood samples were collected with informed consent from CLL patients at the Peter MacCallum Cancer Centre and The Royal Melbourne Hospital (Melbourne, Australia) under ethics approval of the respective institution’s human research ethics committee and in accordance with the Declaration of Helsinki. Peripheral blood mononuclear cells (PBMC) were isolated using Ficoll-Paque plus (GE Healthcare, Chicago, IL) density gradient separation and cryopreserved until required. Peripheral blood samples from age matched Healthy Donor were obtained from the Australian Red Cross Blood Service with ethics approval from the Melbourne Health Human Research Ethics Committee.

### Cell culture

In vitro experiments were performed in this study using HD or treatment naive CLL patient PBMC treated with vehicle or BTKi during the experiment, whereas ex vivo experiments were performed using PBMC from patients collected at baseline or after long-term treatment on BTKi to examine the effect of targeted therapies on immune cell profiles and function. For in vitro analyses, PBMC were thawed and cultured at 1.0 × 10^6^ /mL in T cell media (TCM) (Gibco RPMI media 1640, 10% v/v heat inactivated foetal calf serum (FCS), 1% v/v 4-(2-hydroxyethyl)-1-piperazineethanesulfonic acid (HEPES), 1 ×  Gibco GlutaMAX, 1 ×  Gibco MEM non-essential amino acids, 1 mM Gibco sodium pyruvate, 100 U/mL Penicillin/Streptomycin (all from Thermo Fisher Scientific, Watham, MA) and 50 mM 2-Mercaptoethanol (2ME) (Sigma Aldrich, St Louis, MO)) supplemented with 1 µM ibrutinib (Selleck Chemicals), 1 µM zanubrutinib (BGB-3111, Obtained under a Materials Transfer Agreement with BeiGene Co. Ltd.) or 1 µM acalabrutinib (Selleck Chemicals), or 0.5% DMSO (Sigma Aldrich) vehicle control at 37 °C, 5% CO_2_ for 18 h prior to use in experiments. For ex vivo analysis, PBMC were thawed and plated at 1.0 × 10^6^ /mL in TCM, and used immediately. For all flow cytometry based assays, samples were acquired on a BD LSRFortessa Flow Cytometer (BD Biosciences). Analysis was performed using FlowJo software v10.7.1 (BD Biosciences).

### Degranulation assay

For both the in vitro and ex vivo studies PBMC were activated for four hours with CD2/CD3/CD28 T cell activation beads (Miltenyi Biotec, Cologne, Germany) in the presence of GolgiStop and GolgiPlug protein transport inhibitors (BD Bioscience, Franklin Lakes, NJ), and anti-human CD107a APC conjugated antibody (clone H4A3, BD Bioscience). Cell surface marker staining was performed for the in vitro studies using LIVE/DEAD Aqua fixable dead cell stain (Thermo Fisher Scientific), CD4 APC Cy7 (clone RPA-T4, BD Bioscience), CD3 BV785 (clone OKT3, BioLegend, San Diego, CA) CD8α BV650 (clone RPA-T8, BioLegend), and IFNγ PE (clone B27, BD Biosciences). Cell surface marker staining was performed for the ex vivo study using LIVE/DEAD Aqua fixable dead cell stain, CD4 BUV395 (clone SK3, BD Biosciences), CD3 BUV496 (clone UCHT1, BD Biosciences) and CD8α BUV805 (clone SK1, BD Biosciences). Surface and intracellular cytokine staining was performed using the Cytofix/Cytoperm kit (BD Biosciences) according to manufacturer’s instructions.

### T cell proliferation assay and phenotypic analysis

PBMC were stained with 5 μM CellTrace violet (CTV, Thermo Fisher Scientific) according to manufacturer’s instructions and stimulated with CD2/CD3/CD28 T cell activation beads. For in vitro studies, on days 3 and 5 half of the media was removed from the culture, and 20 IU/mL rhIL-2, and 1 µM BTKi or 0.5% DMSO added in TCM. Cells were harvested on days 3, 5, and 7 for T cell proliferation and cytokine production analysis. For ex vivo studies, on day 3 half of the media was removed from the culture and 20 IU/mL rhIL-2 in TCM was added. Cells were harvested on day 5 for T cell proliferation analysis. Cell surface marker staining was performed using LIVE/DEAD Aqua fixable dead cell stain, CD19 PECy7 (clone SJ25-C1, BD Biosciences), CD4 BUV395 (clone SK3, BD Biosciences), CD3 BUV496 (clone UCHT1, BD Biosciences), CD8 BUV805 (clone SK1, BD Biosciences), CD27 FITC (clone M-T271, BD Biosciences), CD45RA PerCPCy5.5 (clone HI100, Thermo Fisher Scientific), LAG3 AF647 (clone T47-530, BD Bioscience), PD-1 BV786 (clone EH12.1, BD Bioscience), and TIM3 PE (clone 7D3, BD Bioscience). The flow cytometry panel described above was also used for phenotypic analysis of freshly thawed PBMC (Additional file 1: Figure S1).

Proliferation of CD4 + and CD8 + T cells was measured using FlowJo (BD Bioscience) cell proliferation analysis to identify up to 9 generations of dividing cells based on CTV intensity. Generation 0 represented undivided cells, and generation 9 represented cells that had undergone at least 8 divisions. The percentage of cells in generations 0, 1, and 2 was added to give a combined total percentage of cells in generations 0–2, representing undivided and newly dividing cells (Additional file 1: Figure S2). The median fluorescence intensity (MFI) of PD-1, TIM3 and LAG3 expression was calculated for individual generations of dividing CD4 + and CD8 + T cells.

### Cytokine bead array

PBMC were stimulated with CD2/CD3/CD28 T cell activation beads for 24 h to 7 days, and tissue culture media collected. The BD Cytometric Bead Array (CBA) Human Th1/Th2/Th17 cytokine kit (BD Biosciences) was used to quantify the concentration of IL-2, IL-4, IL-6, IL-10, IL17A, IFNγ and TNFα, according to the manufacturer’s instructions. The sensitivity of the assay was 5000 pg/mL and therefore any readings that fell above this level were censored to 5000 pg/mL. CBA analysis was performed using FCAP Array v3.0 software (BD Biosciences).

### Statistical analysis

Prism V9.0 (GraphPad) was used to perform statistical analysis in this study. For the in vitro experiments, statistical analysis was performed using Mixed effects analysis with Tukey–Kramer test for multiple comparisons for the degranulation and ICS experiments; RM one-way ANOVA with Fisher’s LSD for cytokine production; Friedman test, Dunn’s multiple comparisons for exhaustion marker expression; and Two-way ANOVA with Fisher’s LSD multiple comparisons for CTV T cell generation analysis. For the ex vivo experiments, statistical analysis was performed using Mann–Whitney unpaired T tests between the Healthy Donor and CLL patient samples, and Wilcoxon matched pairs T test between the baseline and long-term treatment CLL patient samples. *p < 0.05, **p < 0.01, ***p < 0.001, ****p < 0.0001.

## Results

### T cells improve in number and function after long-term BTKi exposure

**Fig. 1:**
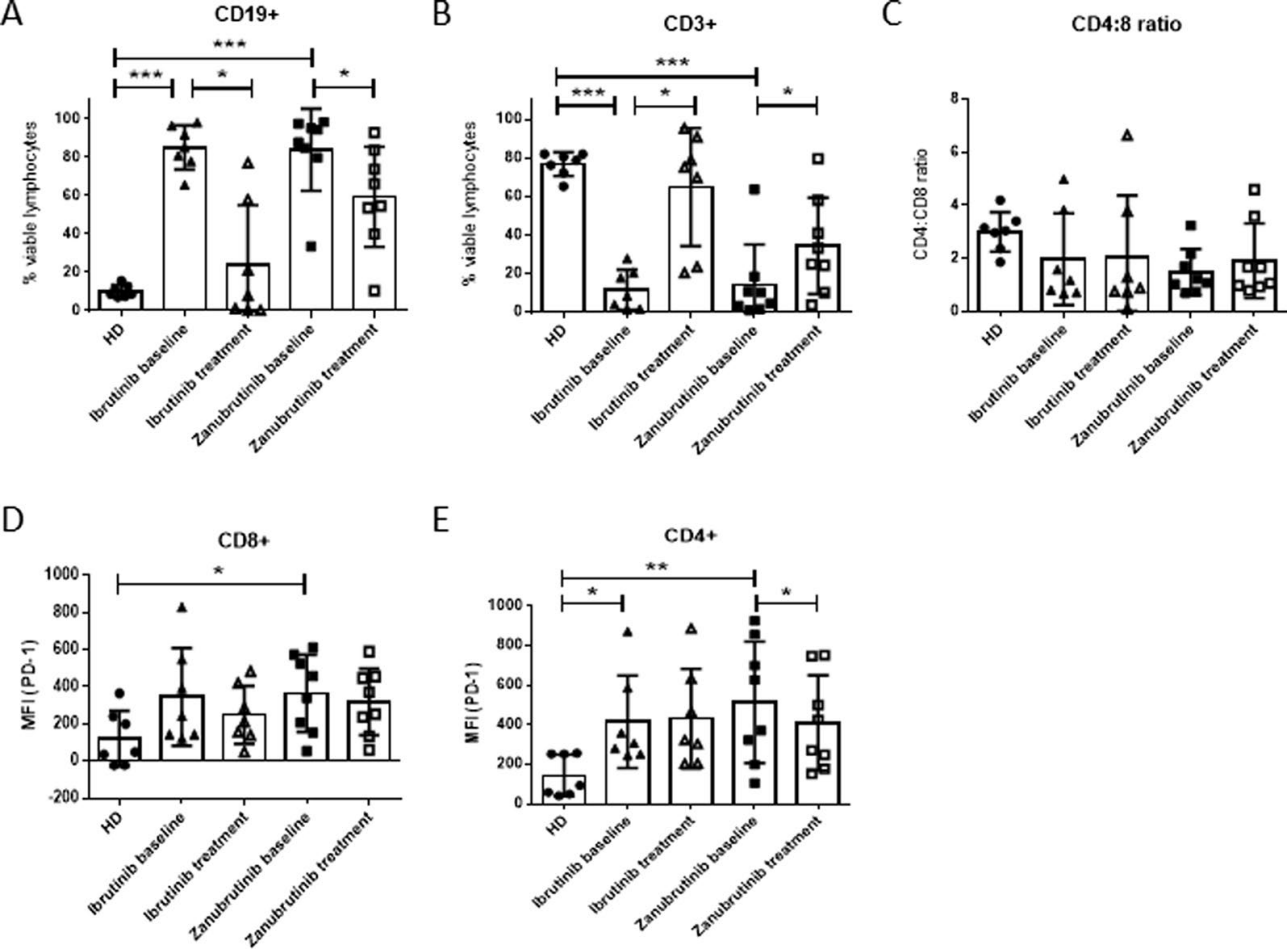
Long-term BTKi treatment normalises lymphocyte subset frequency and reduces PD-1 expression. Frequency of CD19 + B cells (**A**), CD3 + T cells (**B**) and CD4:CD8 ratio (**C**) in PBMC from CLL patients collected prior to treatment (baseline), after long-term treatment of ibrutinib (n = 7) or zanubrutinib (n = 8) and age-matched healthy donors (HD, n = 7). PD-1 median fluorescence intensity (MFI) on CD8 + (**D**) and CD4 + (**E**) T cells

In order to examine the impact of BTKi on T cell function in CLL, peripheral blood samples taken at baseline and following long-term treatment (12–24 months) were analysed. CLL patients treated with BTKi showed a significant reduction in the frequency of circulating CD19^+^ B cells, consistent with the clinical responses observed following long-term ibrutinib or zanubrutinib treatment (Fig. 1A), however most patients still had detectable tumour cells. As described by others [23, 24], a commensurate increase in the percentage of CD3 + T cells following long-term BTKi therapy was observed (Fig. 1B). This was not associated with a change in CD4:CD8 T ratio (Fig. 1C), nor an alteration in the frequency of memory subsets in CD8^+^ T cells (Additional file 1: Figure S3A–D). In contrast, CLL patients had low frequencies of CD4^+^ naïve T cells compared to healthy donors, which significantly increased after long-term zanubrutinib treatment (Additional file 1: Figure S4A–D). Exhaustion marker profiling confirmed that T cells isolated from CLL patients prior to BTKi treatment had increased expression of PD-1 on CD4^+^ and CD8^+^ T cells compared with healthy donors (Fig. 1D, E) which significantly decreased in CD4 + T cells after long-term zanubrutinib treatment.

**Fig. 2:**
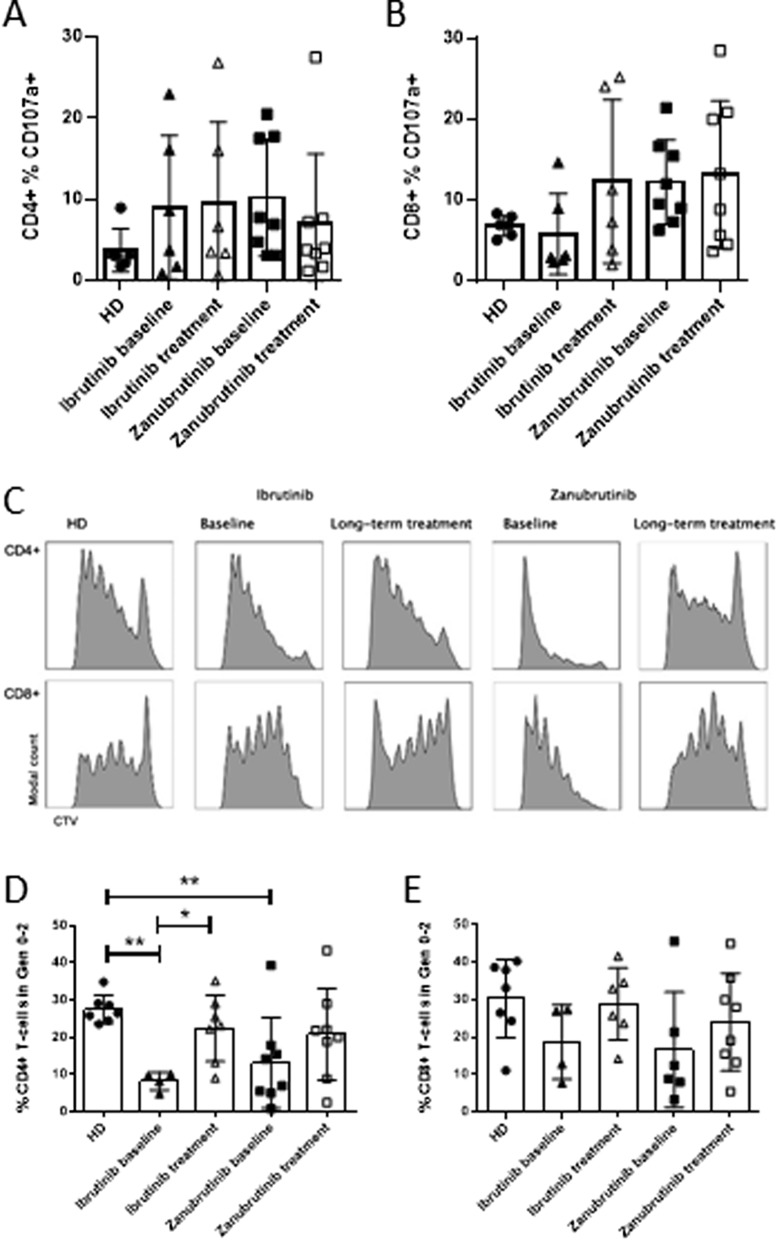
Long-term BTKi treatment normalises abnormal proliferation in CD4 + T cells. **A**, **B** 4 h degranulation of CD4 + and CD8 + T cells at baseline or after long-term treatment of ibrutinib (n = 7) or zanubrutinib (n = 8) compared to Healthy Donor (HD, n = 7). 5 day proliferation assay representative CD8 + and CD4 + T cell CTV proliferation histograms (**C**), and summary of CD4 + and CD8 + T cells in generations 0–2 (D-E)

Ex vivo T cell assays indicated that there were no effects of long-term BTKi treatment on the ability of CD4^+^ or CD8^+^ T cells to degranulate (Fig. 2A, B). This suggested that functional analysis using degranulation may provide an inaccurate measure of drug-induced phenotypic changes in T cell behaviour. We therefore investigated the impact of drug therapy on the ability of T cells to proliferate. Compared to healthy donor T cells, the baseline samples from CLL patients showed increased proliferation resulting in a decreased percentage of cells in early generations (generations 0–2) for CD4^+^ T cells which was corrected to healthy donor levels with ibrutinib treatment (Fig. 2C, D). A similar trend was seen in CD4 + T cells in patients treated with zanubrutinib and in CD8 + T cells treated with either BTKi however this did not reach statistical significance (Fig. 2D, E). Collectively, this data suggests that long-term BTKi exposure results in a reduction in hyperproliferative T cell responses, and may contribute to normalisation of T cell phenotype independent of off-target ITK inhibition.

### Short-term ibrutinib treatment impairs T cell function in vitro

**Fig. 3:**
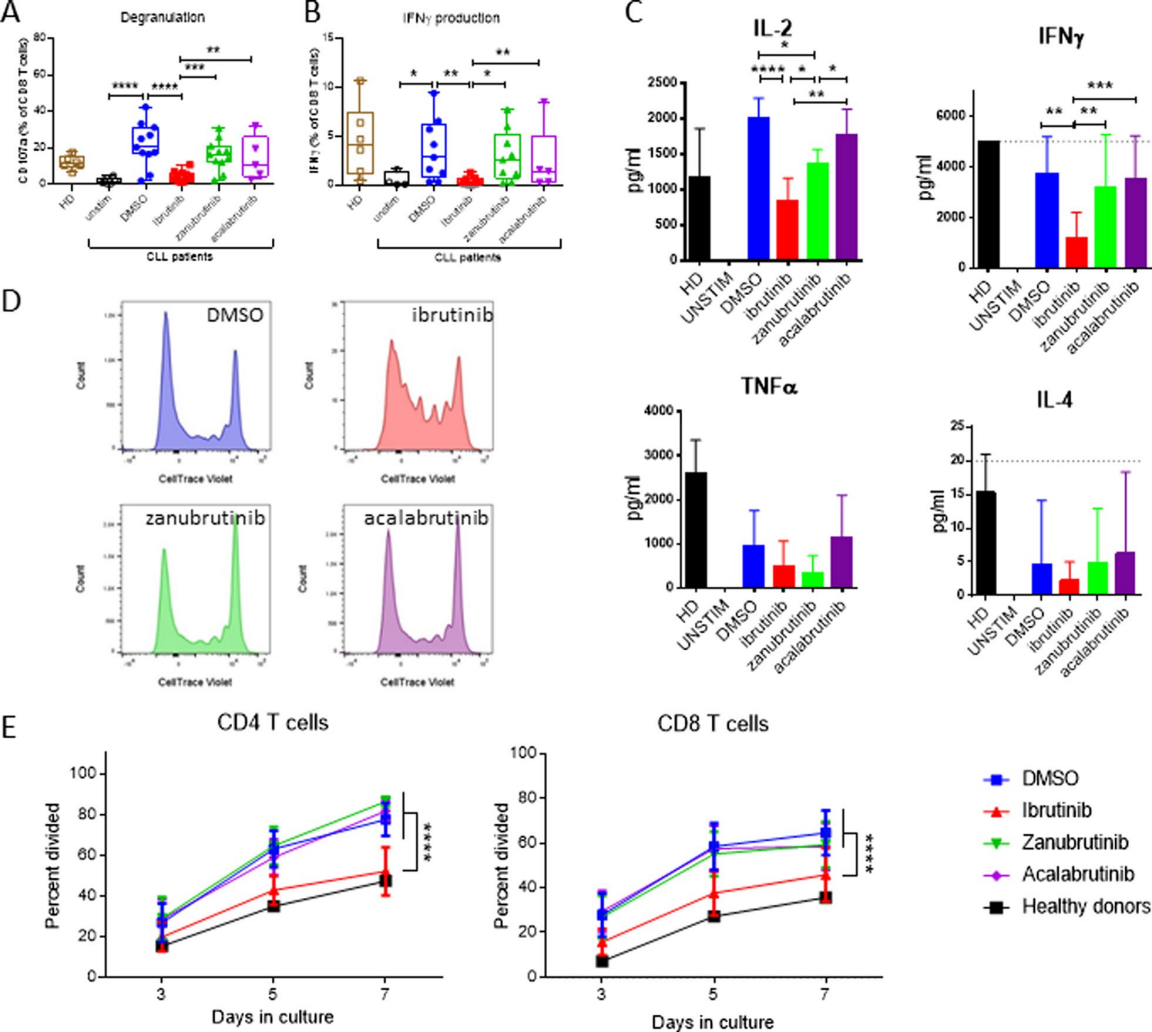
Ibrutinib inhibits T cell degranulation, cytokine release and proliferation of CLL patient T cells in vitro. CD8 + T cell degranulation (**A**) and intracellular IFNγ production (**B**) in PBMC from Healthy Donor (HD, n = 6) or treatment naïve CLL patients (n = 11) incubated with BTKi, or DMSO vehicle control for 18 h prior to activation with T cell stimulation beads for 4 h. **C** IL-2, IFNγ, TNFα and IL-4 levels in the supernatant after 24 h of T cell activation from HD (n = 4) or treatment naïve CLL patients (n = 4) in the presence of BTKi. **D** Representative CTV dilution histograms after 5 day proliferation assay in the presence of different BTKi treatments. **E** Effect on CD8 + and CD4 + T cell proliferation after 3, 5, and 7 days

To explore the mechanisms leading to the observed recovery of T cell numbers, we sought to determine if the various BTKi altered CD8^+^ T cell effector functions in vitro. It has been previously demonstrated that NK cell degranulation is suppressed by ibrutinib, but not by the more selective BTKi [10, 11, 28]. Therefore, we investigated the impact of short-term in vitro ibrutinib, zanubrutinib or acalabrutinib treatment on T cell function, in treatment naïve CLL patient PBMC. CD8^+^ T cell degranulation was significantly inhibited by ibrutinib, compared to the DMSO vehicle control (Fig. 3A). Intracellular IFNγ accumulation was similarly reduced in ibrutinib treated cultures in both CD8 + and CD4 + T cells (Fig. 3B and Additional file 1: Figure S5). Neither zanubrutinib nor acalabrutinib significantly altered CD8^+^ T cell degranulation or IFNγ expression. Interestingly, ibrutinib treated CLL PBMC showed a similar level of CD8^+^ T cell degranulation as healthy donors. However, ibrutinib also inhibited CD8^+^ T cell degranulation and IFNγ expression in healthy donor cultures (Additional file 1: Figure S6) suggesting that the inhibition of T cell degranulation responses by ibrutinib is due to the inhibition of intrinsic CD8 T cell responses, potentially attributable to ITK inhibition by ibrutinib decreasing TCR signalling, rather than alteration to the extrinsic environment (via BTK inhibition in other PBMC subsets).

To further assess the impact of BTKi on short-term T cell functional responses, a 24-h cytokine release assay demonstrated that in vitro treatment with ibrutinib significantly reduced IL-2 and IFNγ secretion by CLL patient PBMC (Fig. 3C). A similar pattern was observed in healthy donor PBMC, in which ibrutinib, but not the more selective BTK inhibitors, resulted in significantly decreased levels of IL-2, IFNγ and TNFα (Additional file 1: Figure S6). These findings further support the conclusion that inhibition of ITK by ibrutinib significantly alters T cell function, while more specific BTKi do not significantly impact intrinsic T cell responses to TCR mediated stimuli.

Next, we examined the effect of BTKi on T cell proliferation. Over 7 days, only ibrutinib, but neither zanubrutinib nor acalabrutinib, significantly reduced CD4^+^ and CD8^+^ T cell proliferation (Fig. 3D, E). Strikingly, short-term treatment of ibrutinib in CLL patient PBMC reduced abnormally high T cell proliferation rates to that of healthy donor levels. These findings suggest that while ibrutinib treatment diminishes the magnitude of TCR mediated T cell responses, long-term in vivo treatment with BTKi may restore T cell fitness and ability to respond to subsequent T cell activation.

### Short-term ibrutinib treatment impairs T cell proliferation and exhaustion in vitro

**Fig. 4:**
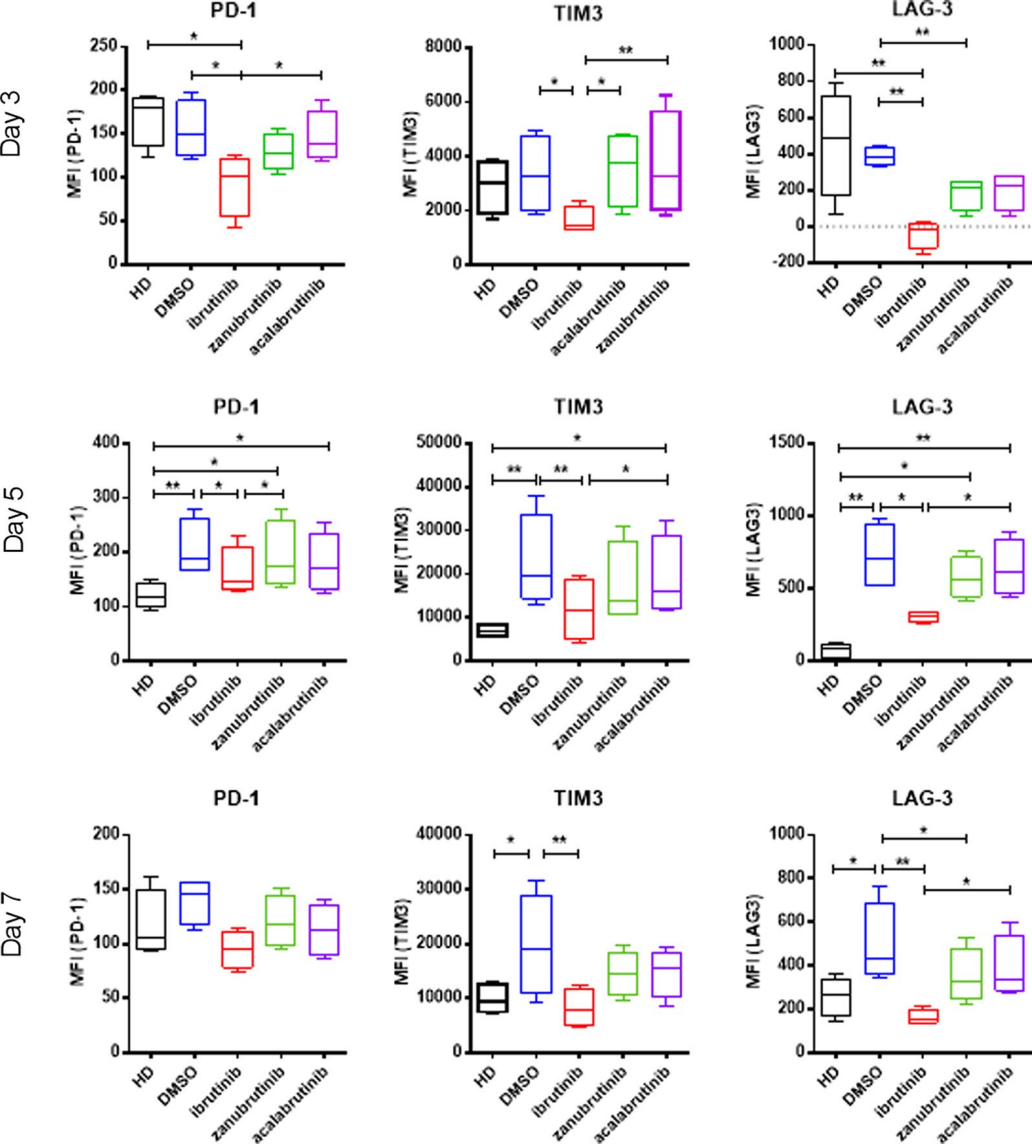
Ibrutinib normalises exhaustion marker expression on CLL patient T cells in vitro. PBMC from Healthy Donor (HD, n = 4) or treatment naive CLL patients (n = 4) were stained with CTV, treated with ibrutinib, zanubrutinib, or acalabrutinib or DMSO, and stimulated with T cell activation beads. Expression of PD-1, TIM3 and LAG3 was determined on days 3, 5 and 7

Patients treated with both ibrutinib and more selective BTKi have significantly reduced expression of co-inhibitory ligands on patient T cells [23, 29]. We therefore sought to understand if this effect was recapitulated after in vitro BTKi treatment of proliferating T cells. Ibrutinib treated CD8^+^ T cells had significantly decreased expression of PD-1, TIM3 and LAG-3 after 3- and 5-days and TIM3 and LAG-3 after 7-days stimulation, while zanubrutinib and acalabrutinib had no significant effect on co-inhibitory ligand expression (Fig. 4). Notably, ibrutinib treated CD8^+^ T cells displayed normalised exhaustion marker expression, similar to the levels observed in age-matched healthy donor T cells. This is the first study to directly compare ibrutinib, acalabrutinib and zanubrutinib treatment on PD-1, TIM3 and LAG3 expression on proliferating T cells from patients with CLL, and highlights the differential impacts of different BTKi on T cell function.

**Fig. 5:**
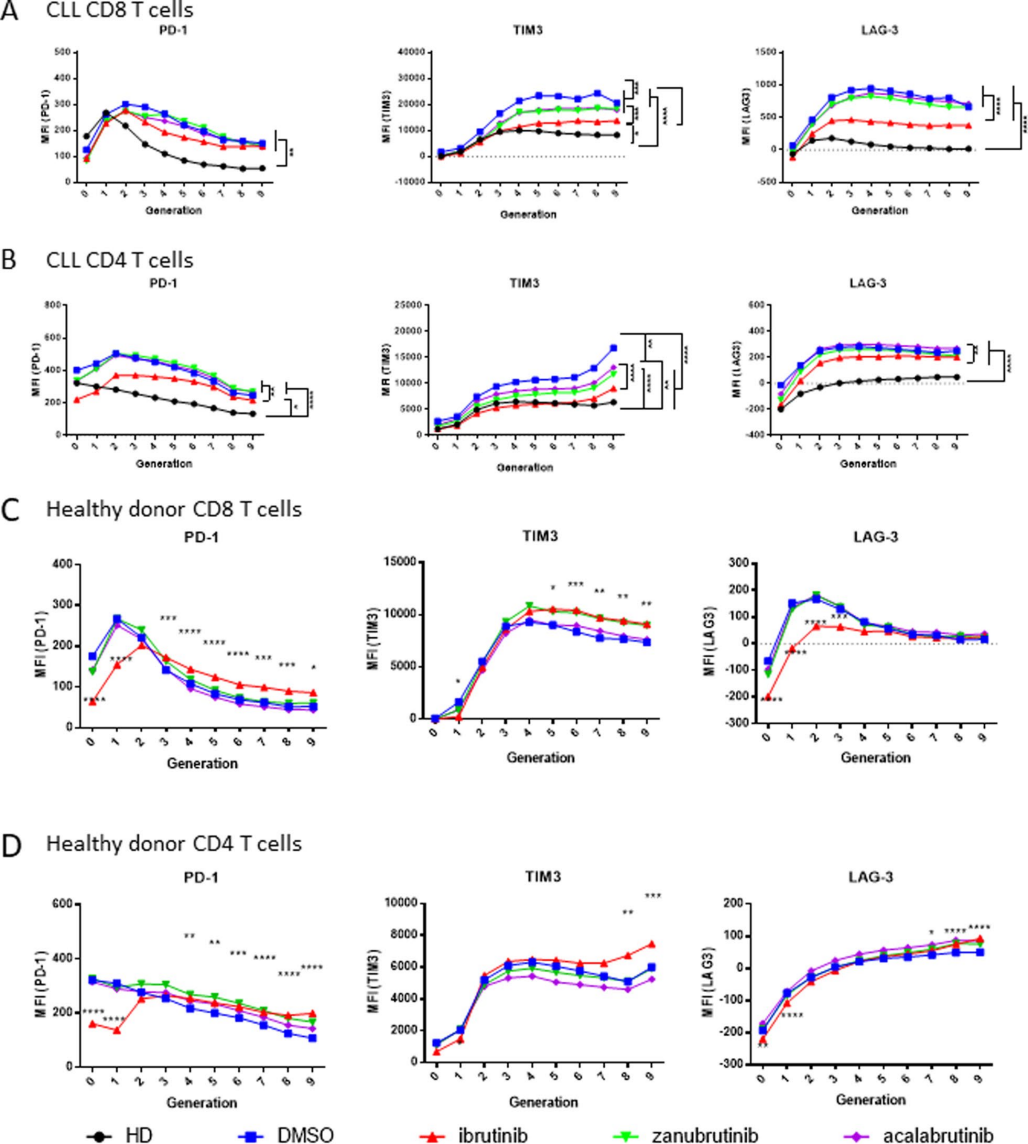
Ibrutinib decreases CD8 T cell proliferation and exhaustion marker expression on CLL patient T cells in vitro. PBMC from Healthy Donor (HD, n = 4) or treatment naive CLL patients (n = 4) were stained with CTV, treated with ibrutinib, zanubrutinib, or acalabrutinib or vehicle control, and stimulated with T cell activation beads. PD-1, TIM3 and LAG3 cell surface expression (MFI) was measured on individual generations of dividing CD8 + and CD4 + T cells from CLL patients (CD8 + (A), CD4 + (B)) and healthy donors (CD8 + (C), CD4 + (D))

Considering that short-term in vitro ibrutinib treatment inhibited T cell proliferation (Fig. 3E), we next explored if changes in the proportion of proliferating cells was associated with decreased exhaustion marker expression. Utilising proliferation gating we compared the expression of exhaustion markers on CD8^+^ and CD4^+^ T cells which had undergone multiple divisions after 5 days stimulation. CD8^+^ T cells treated with ibrutinib had significantly lower expression of TIM3 and LAG3 than control treated counterparts, approaching the levels of expression observed in healthy donor CD8^+^ T cells (Fig. 5A). The effect of ibrutinib treatment on the expression of PD-1, TIM3, and LAG3 on CD4^+^ T cells was less pronounced, however early generations of CD4^+^ T cells had significantly decreased PD-1 expression while later generations had a more normalised expression of TIM3 (Fig. 5B). Notably, zanubrutinib and acalabrutinib did not have a significant effect on PD-1 or LAG3 expression in either CD8^+^ or CD4^+^ T cells, however both decreased the expression of TIM3, albeit to a lesser degree than ibrutinib.

To investigate if the decreased expression of PD-1, TIM3 and LAG3 on CD8^+^ T cells induced by ibrutinib was due to pre-existing CLL patient T cell exhaustion, the effects of BTKi on the proliferation of healthy donor cells was analysed. Healthy donor CD8^+^ T cells treated with ibrutinib exhibited decreased expression of PD-1 and LAG3 compared to vehicle control. However, this effect was restricted to early generations, and in later generations ibrutinib treated CD8^+^ T cells had increased expression of PD-1 and, along with zanubrutinib treated cells, TIM3 (Fig. 5C, D). Together this data suggests that while ibrutinib decreases the expression of exhaustion markers in already exhausted CLL patient T cells, the effect in healthy donor T cells is predominantly in preventing the generation of exhaustion, probably due in part to decreased T cell proliferation.

### Long-term BTKi exposure reduces T cell senescence

**Fig. 6:**
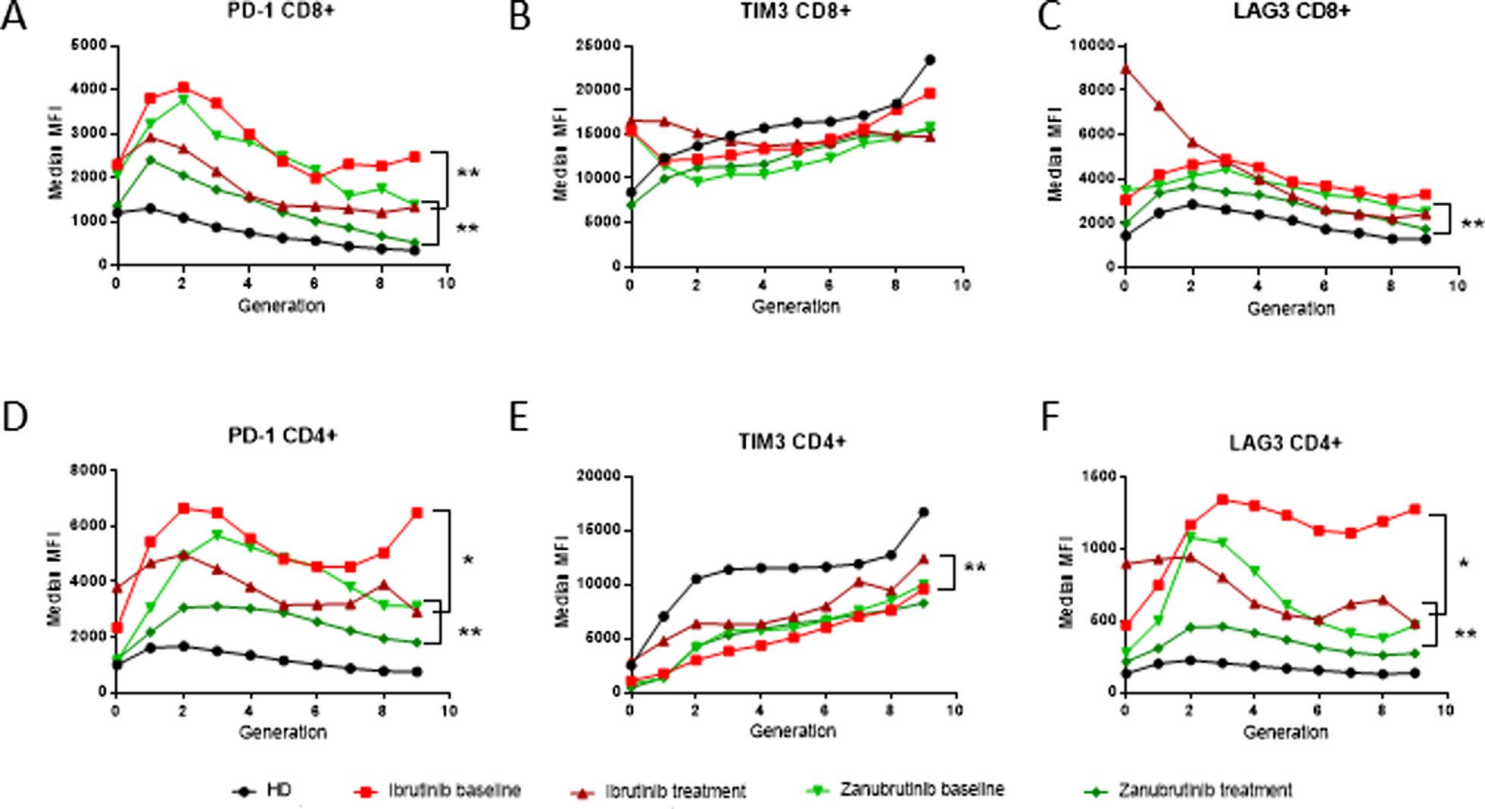
Long-term BTKi treatment normalises CD8 + and CD4 + exhaustion marker expression in dividing T cells. Proliferation of PBMC from CLL patients collected at baseline or after long-term treatment of ibrutinib (n = 7) or zanubrutinib (n = 8), and healthy donor (HD, n = 7). PD-1, TIM3 and LAG3 cell surface expression (MFI) was measured on individual generations of dividing cells (**A**–**F**) after 5 days stimulation. Data was analysed using Mann–Whitney (HD vs patient sample) or Wilcoxon matched-pairs signed rank (baseline vs treatment sample) tests

To confirm that ibrutinib exposure resulted in T cell populations that were less likely to undergo senescence following proliferation, we examined exhaustion marker expression on proliferating T cells collected from CLL patients prior to, and after long-term BTKi exposure. Exhaustion marker analysis on T cells after 5 days of stimulation indicated that CD4^+^ and CD8^+^ T cells from CLL patient baseline samples had elevated expression of PD-1 compared to healthy donor T cells (Additional file 1: Figure S7). After long-term treatment with zanubrutinib, expression of PD-1 significantly decreased on both CD4^+^ and CD8^+^ T cells, towards healthy donor levels. Generational gating was used on individual CD4^+^ and CD8^+^ T cell populations to dissect out the change in exhaustion marker expression with cell division. The expression of PD-1 and LAG3 was highly elevated in the multiply divided T cells from baseline samples collected from CLL patients, compared to healthy donor T cells (Fig. 6), indicative of the formation of an exhausted pool of T cells. Long-term treatment of CLL patients with either ibrutinib or zanubrutinib resulted in a significant decrease of PD-1 and LAG3 expression in dividing T cells, and an increase of TIM3 expression, thus resulting in a T cell population with a less exhausted phenotype despite persistent circulating tumour burden and ongoing antigen stimulation (Fig. 1A).

## Discussion

The development of BTKi drug therapy has substantially improved the efficacy and reduced the toxicity of CLL treatment. The use of BTKi appears to result in a clinical improvement in immunity with less observed opportunistic infections compared to historical cohorts treated with conventional chemotherapy. Indeed, others have recently shown that these clinical observations are supported by a measured increase in CD4^+^ and CD8^+^ T cell numbers and a lowered expression of exhaustion markers, particularly PD-1 in patients treated with ibrutinib, but not the more selective BTKi drug acalabrutinib [23]. In contrast, total CD4^+^ and CD8^+^ T cells have been reported to be unchanged in patients treated with zanubrutinib but these cells exhibit reduced PD-1 expression [30]. Understanding the mechanisms underpinning this apparent improvement in T cell fitness is important, not only because of its wider implications for supportive care of patients with CLL, but also for the optimal timing of immunotherapy in CLL and other low grade B cell malignancies. Whilst the impact of cellular and T cell-engaging immunotherapy in these diseases has initially been disappointing, their role is being re-explored in the context of earlier application or in combination with initial non-chemotherapeutic debulking strategies, including in combination with BTKi. Both approaches may result in enhanced efficacy of immunotherapy through exploiting a less exhausted and fitter effector T cell population, which has been recently identified as a critical determinant of immunotherapy responses [31].

Importantly, the exploration of the impact of BTKi on T cell function may be misleading if interpretation is solely reliant on short-term in vitro analyses. As we and others [23] have demonstrated, in vitro ibrutinib treatment results in suppression of key T cell functions from both CLL and healthy donors including TCR-mediated proliferation, cytokine production and degranulation (Fig. 3 and Additional file 1: Figures S5, S6), the last of which is a reliable surrogate maker for T cell cytotoxic capacity [32]. However, given the baseline abnormal hyperproliferative response seen in CLL T cells, treatment with ibrutinib, but not other BTKi, actually results in a reduction in proliferation to that seen in healthy donor cells (Fig. 3D). This may explain the fact that despite these apparently suppressive effects on T cell function, we and others have consistently observed an increase in CD3^+^ T cell numbers following long-term treatment with ibrutinib but to a much lesser extent with acalabrutinib [23] or zanubrutinib (Fig. 1). Whilst the frequency of T cells in the blood was increased after long-term BTKi therapy as a result of the reduction of CLL burden in the patients we analysed, none had complete clearance of circulating tumour cells despite many months of treatment. Given this, we feel that our findings represent a direct effect of BTKi therapy onthe function and phenotype of T cells rather than as a function of tumor debulking alone.

We wished to explore further these apparently contradictory findings of short-term in vitro suppression of T cell function and their long-term recovery following exposure to ibrutinib. We have been able to assess the impact of ibrutinib, acalabrutinib and zanubrutinib on the T cell phenotype and function in vitro and additionally examine the effects of ibrutinib and zanubrutinib on two cohorts of patients treated at our centre.

Our findings indicate that ibrutinib-induced suppression of T cell proliferation can prevent T cell exhaustion, and contribute to an increased CD3^+^ T cell pool. In an analysis of the expression of the exhaustion markers PD-1, TIM3 and LAG3, we identified that the expression of all three decreased on CD4^+^ and CD8^+^ T cells from untreated patients with CLL after three days of culture in the presence of ibrutinib, but not acalabrutinib or zanubrutinib. Others have suggested that ibrutinib-induced suppression of PD-1 may indicate protection of T cells from activation-induced cell death [23]. In our analysis, a longitudinal time course of in vitro culture indicated that PD-1 suppression by ibrutinib may be transient, whereas suppression of LAG3 and TIM3 is more durable (Fig. 4). As indicated previously these effects appear to be ibrutinib-specific. The development of an exhausted T cell phenotype is most likely to occur in the setting of chronic inflammation-induced T cell proliferation including that induced by aging, chronic infection and malignancy, and may underpin the T cell immunosuppression associated with these conditions [3]. We reasoned that suppression of abnormal T cell proliferation should also be associated with avoidance of exhaustion potentially independent of tumour control. Indeed, in an analysis of proliferation, ibrutinib reduced the proportion of late generation CD8^+^ T cells following CD3/CD28 bead stimulation, which showed a significantly lower expression of the exhaustion markers LAG3 and TIM3 (Figs. 4 and 5). A lesser effect was seen with acalabrutinib and zanubrutinib and with all three drugs in CD4^+^ T cells (Fig. 4). This same pattern was seen in the T cells examined from patients treated with ibrutinib, and to a lesser extent with zanubrutinib for 12–24 months (Fig. 6) indicating that BTKi therapy protected the T cell population present at the time of treatment initiation from the development of an exhausted phenotype. This is further highlighted by the observation of retention of normal degranulation following long-term ibrutinib treatment (Fig. 2).

Collectively our data show that ibrutinib can induce reduction in critical T cell functions when assessed in short-term in vitro cultures. These findings belie the observations of an improved T cell repertoire and improved clinical immune function after long-term therapy with ibrutinib. This improvement stems from the ability of ibrutinib to protect T cells from the development of an exhausted phenotype that follows proliferation and maintenance of an enriched naïve T cell population. These effects seem to require the off-target suppression of ITK by ibrutinib as the effects are not observed following acalabrutinib or zanubrutinib therapy (Fig. 3). In this sense the less specific effects of ibrutinib are important for the observed T cell effects and may underpin the recent findings that ibrutinib improves the production of CAR-T cellsin patients with CLL previously treated with ibrutinib [26] and that ibrutinib supplementation during CAR-T production results in a greater cell yield with a more naïve-like phenotype and decreased expression of exhaustion markers [33]. Indeed, based on these findings a number of trials have been initiated utilising BTKi in combination with CAR-T administration in MCL (NCT04234061) and CLL (NCT03960840). Faced with a choice of BTKi, and with an opportunity to avoid the off-target side effects associated with ibrutinib, the use of more specific BTKi therapy may inadvertently miss out on the opportunities for improvement in T cell fitness. In this context, ibrutinib may be increasingly considered an immunomodulatory drug that can be applied outside of B cell malignancies. Indeed, the immunomodulatory effect of ibrutinib on ‘off target’ immune cells is being investigated for the treatment of metabolic inflammatory disorders such as Type 2 Diabetes [34].

Overall, our findings indicate that a programmatic approach to CLL and other B cell malignancies should be considered, whereby ibrutinib therapy for both disease control and improved T cell fitness is followed immediately by immunotherapy in the form of either CAR-T cell therapy or BiTE therapy. This two-pronged approach will permit clearance of residual tumour cells prior to relapse and exploit the more functionally normal, and therefore more fit, T cell repertoire in order to achieve deeper and more durable responses and potentially a cure in these previously incurable malignancies.

## Supplementary Information

**Additional file 1: Figure S1.** Flow cytometry gating strategy. PBMC from HD or CLL patients were gated on single cells, lymphocytes, live cells, and CD19 + B or CD3 + T cells. The memory phenotype of CD8 + or CD4 + T cells was measured using Naïve (N, CD27 + CD45RA +), Central memory (CM, CD27 + CD45RA-), effector memory (EM, CD27-CD45RA-), and terminally differentiated effector memory (TEMRA, CD27-CD45RA +) subsets. CD4 + and CD8 + T cells were also tested for the expression of PD-1, TIM3 and LAG3. Flow gating in the bottom row shows representative CD8 + T cell subsets. **Figure S2.** Generation 0–2 cell proliferation analysis. Proliferation of CD4 + and CD8 + T cells was measured using FlowJo cell proliferation analysis to identify 9 generations of dividing cells. Generation 0 represents undivided cells, and generation 9 represents cells that have undergone at least 8 divisions. The percentage of cells in generations 0, 1, and 2 was added to give a combined total % of cells in generations 0–2, representing undivided or early generation T cells. **Figure S3.** Stable memory phenotype CD8 + T cell subset frequency in CLL patients on long-term BTKi therapy. PBMC from CLL patients collected prior to treatment (baseline), or after long-term treatment of ibrutinib (n = 7) or zanubrutinib (n = 8) were compared to Healthy Donor (HD, n = 7). Naïve (N, CD27 + CD45RA +), Central memory (CM, CD27 + CD45RA-), effector memory (EM, CD27-CD45RA-), and terminally differentiated effector memory (TEMRA, CD27-CD45RA +) cells (A-D) were calculated as a percentage of CD8 + T cells. **Figure S4.** Increased naive CD4 + T cell subset frequency in CLL patients on long-term BTKi therapy. PBMC from CLL patients collected prior to treatment (baseline), or after long-term treatment of ibrutinib (n = 7) or zanubrutinib (n = 8) were compared to Healthy Donor (HD, n = 7). Naïve (N, CD27 + CD45RA +), Central memory (CM, CD27 + CD45RA-), effector memory (EM, CD27-CD45RA-), and terminally differentiated effector memory (TEMRA, CD27-CD45RA +) cells (A-D) were calculated as a percentage of CD4 + T cells. **Figure S5.** Ibrutinib inhibits cytokine release in CD4 + T cells from CLL patients in vitro. PBMC from untreated CLL patients (n = 5) were incubated with 1 μM ibrutinib, zanubrutinib, or acalabrutinib, or DMSO vehicle control for 18 h prior to activation with T cell stimulation beads for 4 h. CD4 + T cell intracellular IFNγ production is shown, and statistical analysis was performed using mixed effects analysis with Tukey–Kramer test for multiple comparisons. **Figure S6.** Ibrutinib inhibits T cell cytotoxicity, cytokine release and proliferation of healthy donor T cells in vitro. PBMC from Healthy Donor (HD, n = 6) were incubated with 1 μM ibrutinib, zanubrutinib, or acalabrutinib, or DMSO vehicle control for 18 h prior to activation with T cell stimulation beads for 4 h. CD8 + T cell degranulation (A) intracellular IFNγ production (B) are shown, and statistical analysis was performed using mixed effects analysis with Tukey–Kramer test for multiple comparisons. IL-2, IFNγ, TNFα and IL-4 levels in the supernatant were measured after 24 h of T cell activation bead stimulation of PBMC from HD (n = 4) in the presence of BTKi (C). The sensitivity of the assay was limited to 5000 pg/ml. Data was analysed using RM one-way ANOVA with Fisher’s LSD. **Figure S7.** Long-term BTKi treatment normalises CD8 + and CD4 + exhaustion marker expression in dividing T cells. Proliferation of PBMC from CLL patients collected at baseline or after long-term treatment of ibrutinib (n = 7) or zanubrutinib (n = 8), and Healthy Donor (HD, n = 7). PD-1, TIM3 and LAG3 cell surface expression (MFI) was measured on total CD8 + and CD4 + T cells (A-F). Data was analysed using Mann–Whitney (HD vs patient sample) or Wilcoxon matched-pairs signed rank (baseline vs treatment sample) tests.

**Additional file 2: Table S1.** CLL patient and healthy donor characteristics.

## Data Availability

The datasets used and/or analysed during the current study are available from the corresponding author on reasonable request and approval from the relevant ethics committees.

## Abbreviations

BiTE: Bispecific T cell engager
CAR: Chimeric antigen receptor
CLL: Chronic lymphocytic leukaemia
BTKi: Bruton’s Tyrosine Kinase inhibitor
PD-1: Program cell death molecule-1
CTLA-4: Cytotoxic T lymphocyte antigen-4
ITK: IL-2 inducible T cell kinase
MCL: Mantle cell lymphoma
PBMC: Peripheral blood mononuclear cells
CTV: Cell trace violet
CBA: Cytokine bead array
