# Supplementary material for: Ibrutinib protects T cells in patients with CLL from proliferation-induced senescence
Source: J Transl Med. 2021 Nov 22;19:473. doi: 10.1186/s12967-021-03136-2 (PMC8609739; doi:10.1186/s12967-021-03136-2)
Supplement: Supplementary file 1 — Additional file 1: Figure S1. Flow cytometry gating strategy. PBMC from HD or CLL patients were gated on single cells, lymphocytes, live cells, and CD19 + B or CD3 + T cells. The memory phenotype of CD8 + or CD4 + T cells was measured using Naïve (N, CD27 + CD45RA +), Central memory (CM, CD27 + CD45RA-), effector memory (EM, CD27-CD45RA-), and terminally differentiated effector memory (TEMRA, CD27-CD45RA +) subsets. CD4 + and CD8 + T cells were also tested for the expression of PD-1, TIM3 and LAG3. Flow gating in the bottom row shows representative CD8 + T cell subsets. Figure S2. Generation 0–2 cell proliferation analysis. Proliferation of CD4 + and CD8 + T cells was measured using FlowJo cell proliferation analysis to identify 9 generations of dividing cells. Generation 0 represents undivided cells, and generation 9 represents cells that have undergone at least 8 divisions. The percentage of cells in generations 0, 1, and 2 was added to give a combined total % of cells in generations 0–2, representing undivided or early generation T cells. Figure S3. Stable memory phenotype CD8 + T cell subset frequency in CLL patients on long-term BTKi therapy. PBMC from CLL patients collected prior to treatment (baseline), or after long-term treatment of ibrutinib (n = 7) or zanubrutinib (n = 8) were compared to Healthy Donor (HD, n = 7). Naïve (N, CD27 + CD45RA +), Central memory (CM, CD27 + CD45RA-), effector memory (EM, CD27-CD45RA-), and terminally differentiated effector memory (TEMRA, CD27-CD45RA +) cells (A-D) were calculated as a percentage of CD8 + T cells. Figure S4. Increased naive CD4 + T cell subset frequency in CLL patients on long-term BTKi therapy. PBMC from CLL patients collected prior to treatment (baseline), or after long-term treatment of ibrutinib (n = 7) or zanubrutinib (n = 8) were compared to Healthy Donor (HD, n = 7). Naïve (N, CD27 + CD45RA +), Central memory (CM, CD27 + CD45RA-), effector memory (EM, CD27-CD45RA-), and terminally differentiated effecto [file 12967_2021_3136_MOESM1_ESM.docx]

**Supplementary Table 1.**

CLL patient and healthy donor characteristics.

| **Sample cohort** | **Number** | **Average age** | **Sex** |
| --- | --- | --- | --- |
| Healthy donor (in vitro study) | 6 | 70.5 +/- 6.3 (62-77) | 2 M /4 F |
| Healthy donor (ex vivo study) | 7 | 69.1 +/- 4.2 (64-77) | 4 M/ 3 F |
| Treatment naive CLL | 11 | 66.2 +/- 8.7 (61-88) | 7 M /4 F |
| Ibrutinib | 7 | 63.1 +/- 11.2 (49-74) | 5 M/ 2 F |
| Zanubrutinib | 8 | 63.9 +/- 11.2 (46-79) | 6 M/ 2 F |


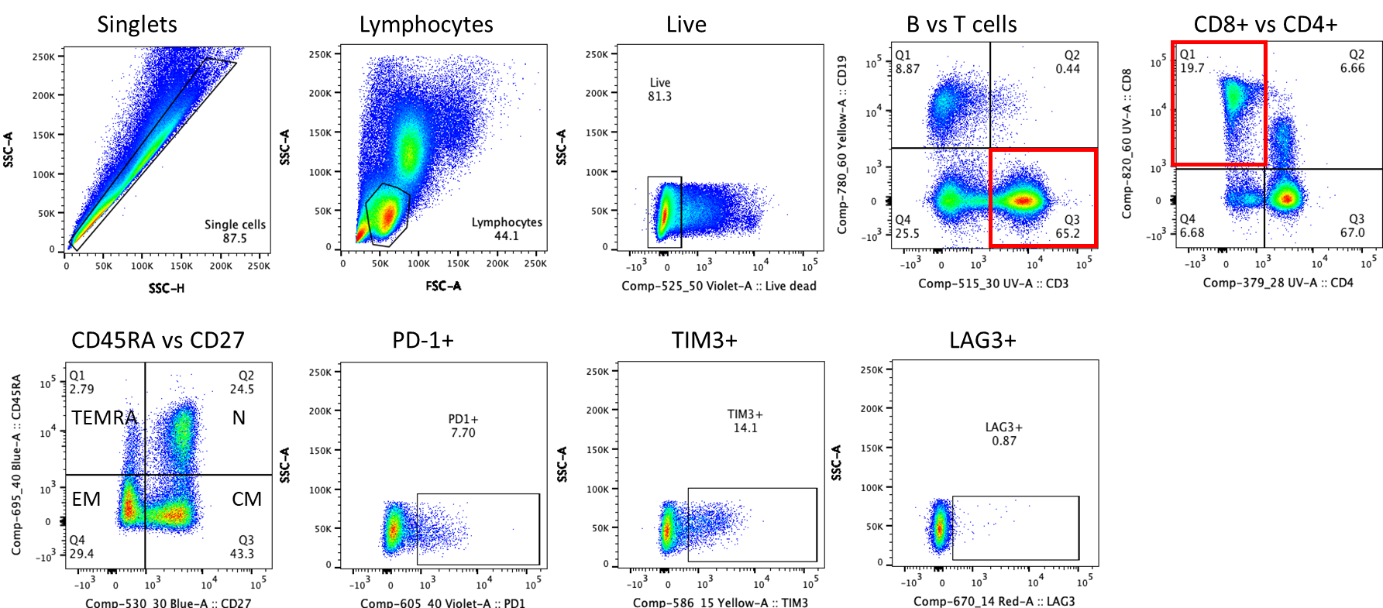


## Supplementary Figure 1: Flow cytometry gating strategy. PBMC from HD or CLL patients were gated on single cells, lymphocytes, live cells, and CD19+ B or CD3+ T cells. The memory phenotype of CD8+ or CD4+ T cells was measured using Naïve (N, CD27+CD45RA+), Central memory (CM, CD27+CD45RA-), effector memory (EM, CD27-CD45RA-), and terminally differentiated effector memory (TEMRA, CD27-CD45RA+) subsets. CD4+ and CD8+ T cells were also tested for the expression of PD-1, TIM3 and LAG3. Flow gating in the bottom row shows representative CD8+ T cell subsets.


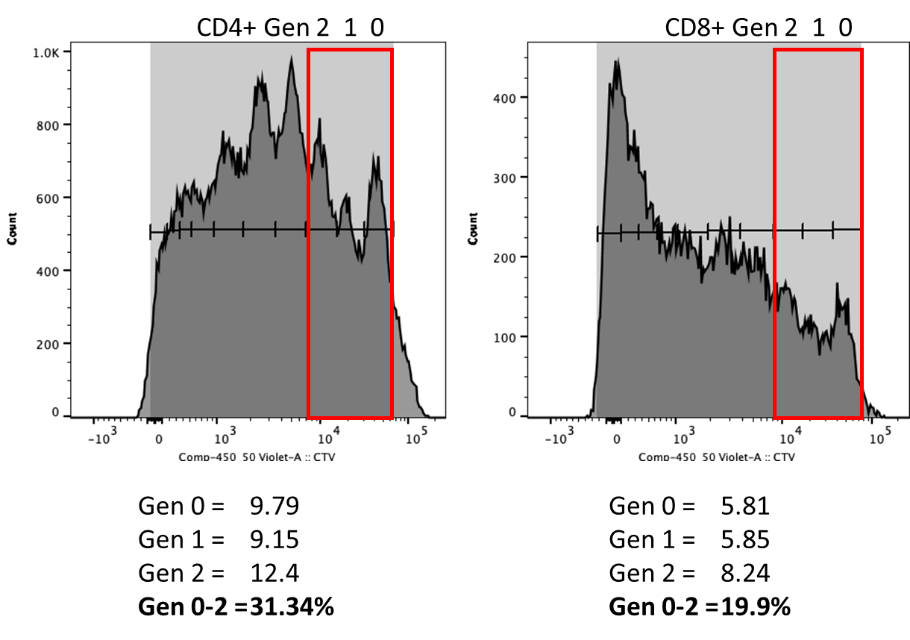


**Supplementary Figure 2: Generation 0-2 cell proliferation analysis.** Proliferation of CD4+ and CD8+ T cells was measured using FlowJo cell proliferation analysis to identify 9 generations of dividing cells. Generation 0 represents undivided cells, and generation 9 represents cells that have undergone at least 8 divisions. The percentage of cells in generations 0, 1, and 2 was added to give a combined total % of cells in generations 0-2, representing undivided or early generation T cells.


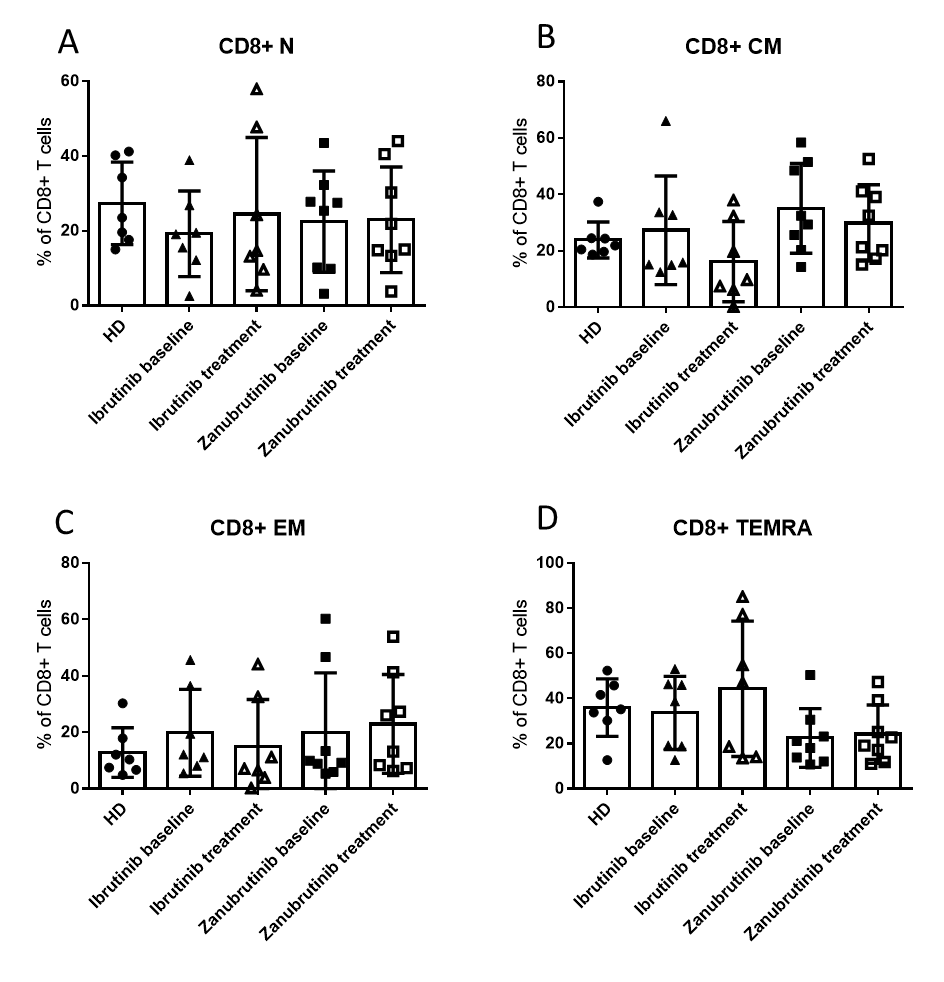


## Supplementary Figure 3: Stable memory phenotype CD8+ T cell subset frequency in CLL patients on long-term BTKi therapy. PBMC from CLL patients collected prior to treatment (baseline), or after long-term treatment of ibrutinib (n=7) or zanubrutinib (n=8) were compared to Healthy Donor (HD, n=7). Naïve (N, CD27+CD45RA+), Central memory (CM, CD27+CD45RA-), effector memory (EM, CD27-CD45RA-), and terminally differentiated effector memory (TEMRA, CD27-CD45RA+) cells (A-D) were calculated as a percentage of CD8+ T cells.


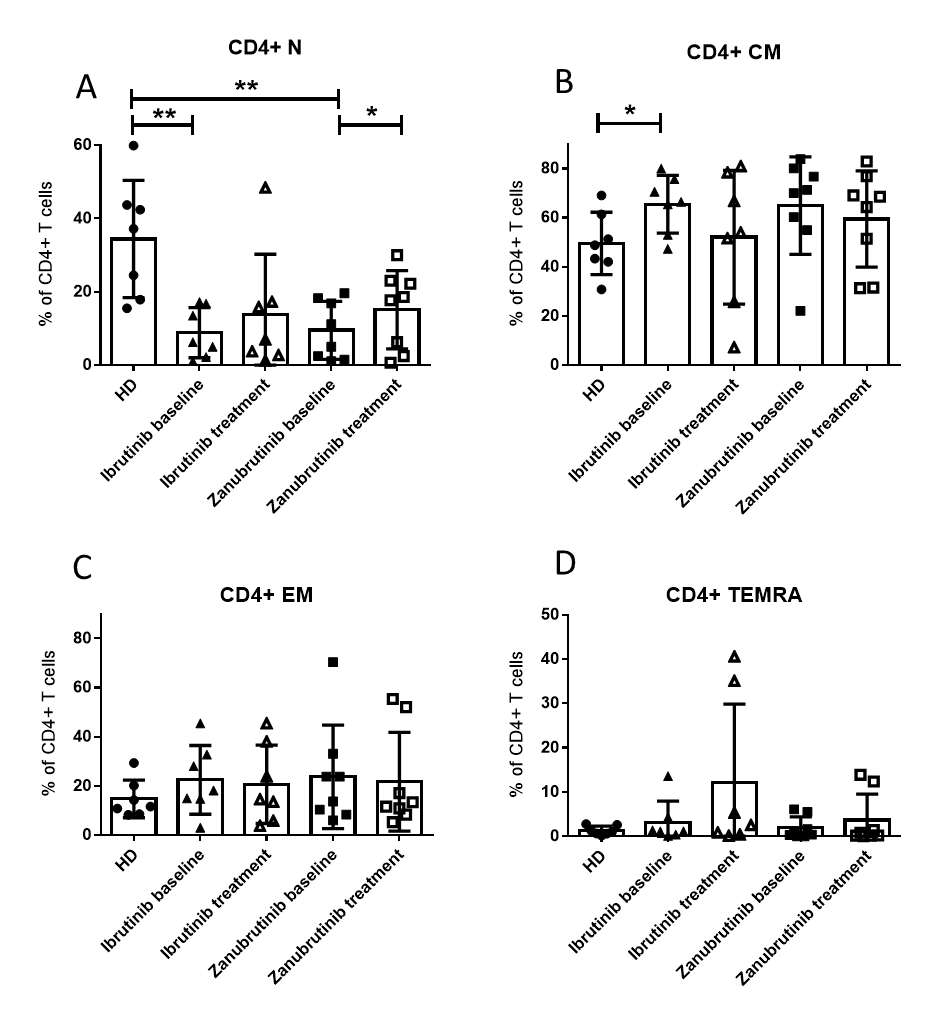


## Supplementary Figure 4: Increased naive CD4+ T cell subset frequency in CLL patients on long-term BTKi therapy. PBMC from CLL patients collected prior to treatment (baseline), or after long-term treatment of ibrutinib (n=7) or zanubrutinib (n=8) were compared to Healthy Donor (HD, n=7). Naïve (N, CD27+CD45RA+), Central memory (CM, CD27+CD45RA-), effector memory (EM, CD27-CD45RA-), and terminally differentiated effector memory (TEMRA, CD27-CD45RA+) cells (A-D) were calculated as a percentage of CD4+ T cells.


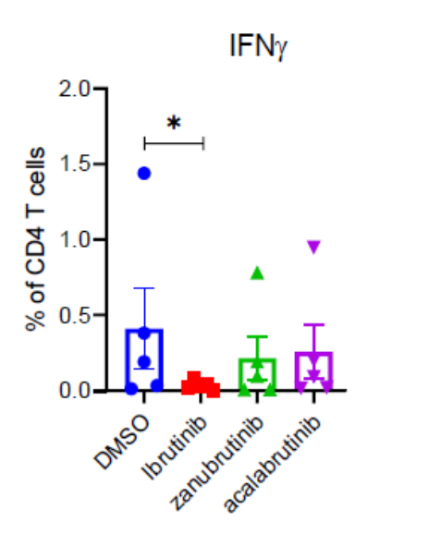


## Supplementary Figure 5. Ibrutinib inhibits cytokine release in CD4+ T cells from CLL patients in vitro. PBMC from untreated CLL patients (n=5) were incubated with 1 μM ibrutinib, zanubrutinib, or acalabrutinib, or DMSO vehicle control for 18 hours prior to activation with T cell stimulation beads for 4 hours. CD4+ T cell intracellular IFNγ production is shown, and statistical analysis was performed using mixed effects analysis with Tukey-Kramer test for multiple comparisons.


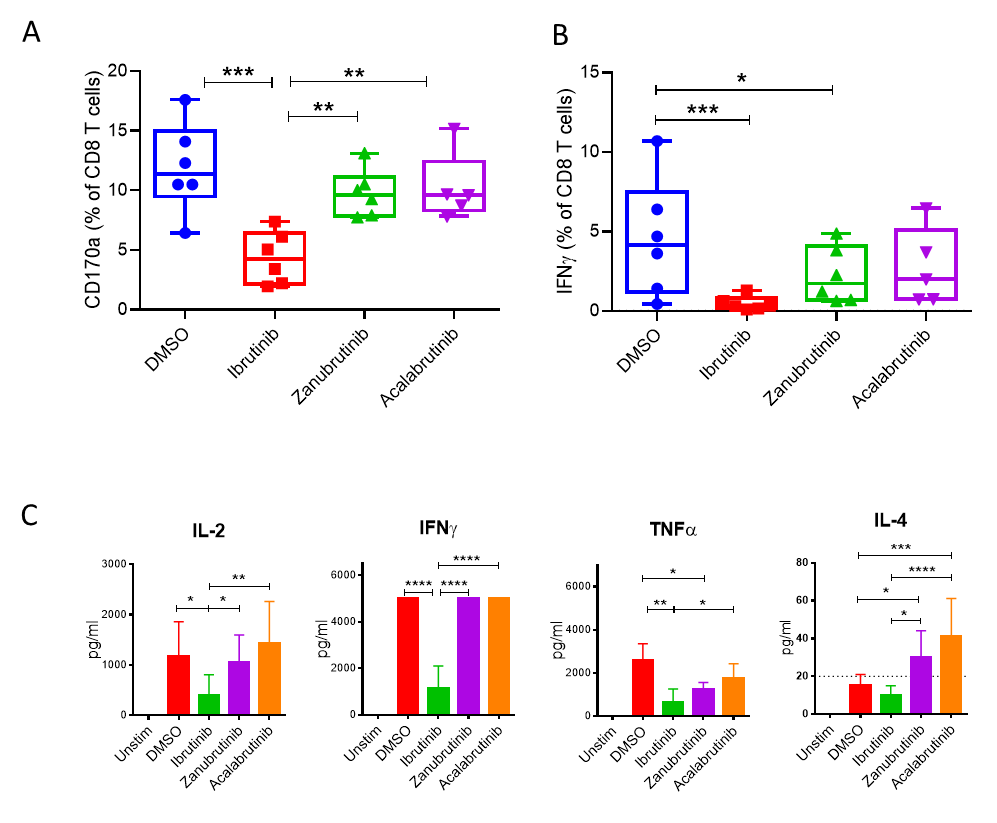


## Supplementary Figure 6. Ibrutinib inhibits T cell cytotoxicity, cytokine release and proliferation of healthy donor T cells in vitro. PBMC from Healthy Donor (HD, n=6) were incubated with 1 μM ibrutinib, zanubrutinib, or acalabrutinib, or DMSO vehicle control for 18 hours prior to activation with T cell stimulation beads for 4 hours. CD8+ T cell degranulation (A) intracellular IFNγ production (B) is shown, and statistical analysis was performed using mixed effects analysis with Tukey-Kramer test for multiple comparisons. IL-2, IFNγ, TNFα and IL-4 levels in the supernatant were measured after 24 hours of T cell activation bead stimulation of PBMC from HD (n=4) in the presence of BTKi (C). The sensitivity of the assay was limited to 5000 pg/ml. Data was analysed using RM one-way ANOVA with Fisher’s LSD.


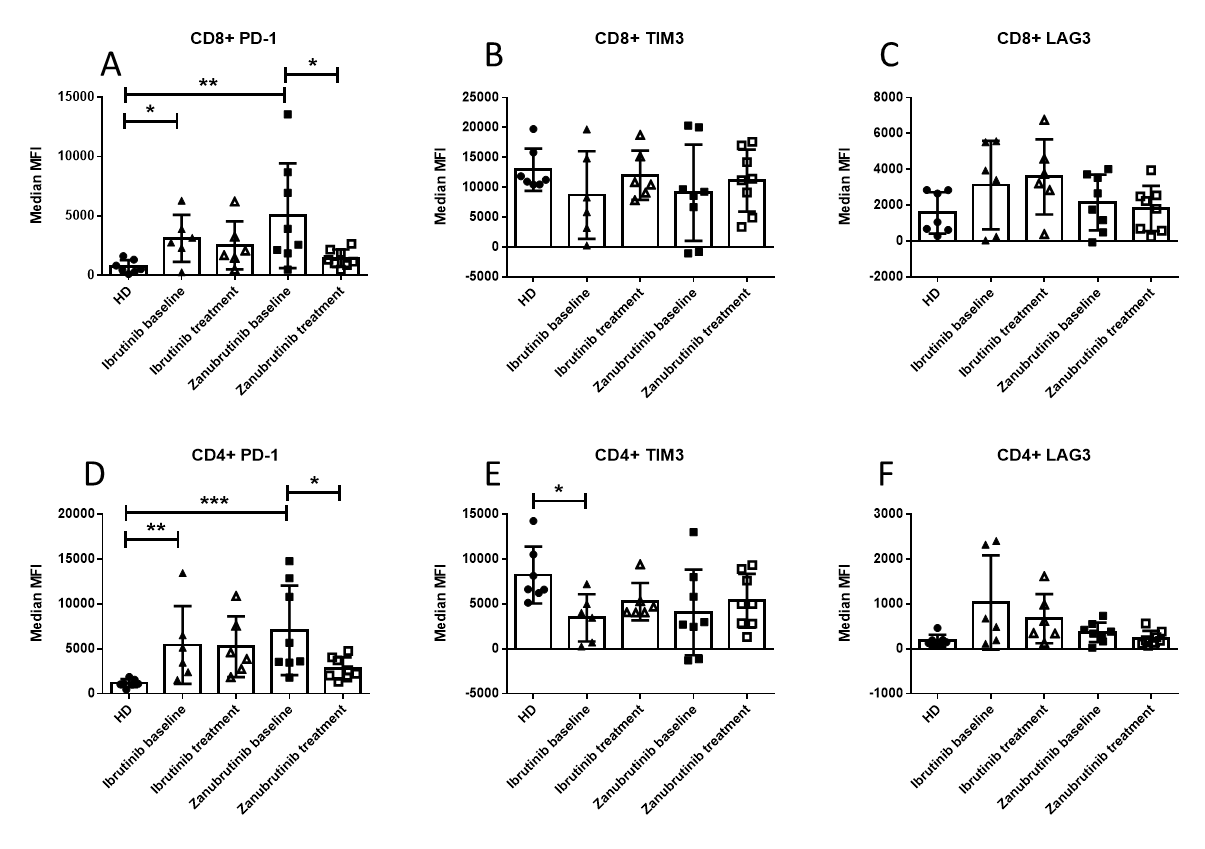


**Supplementary Figure 7: Long-term BTKi treatment normalises CD8+ and CD4+ exhaustion marker expression in dividing T cells.** Proliferation of PBMC from CLL patients collected at baseline or after long-term treatment of ibrutinib (n=7) or zanubrutinib (n=8), and Healthy Donor (HD, n=7). PD-1, TIM3 and LAG3 cell surface expression (median MFI) was measured on total CD8+ and CD4+ T cells after 5 days stimulation (A-F). Data was analysed using Mann-Whitney (HD vs patient sample) or Wilcoxon matched-pairs signed rank (baseline vs treatment sample) tests.
