# Supplementary material for: Ibrutinib protects T cells in patients with CLL from proliferation-induced senescence
Source: J Transl Med. 2021 Nov 22;19:473. doi: 10.1186/s12967-021-03136-2 (PMC8609739; doi:10.1186/s12967-021-03136-2)
Supplement: Supplementary file 2 — Additional file 2: Table S1. CLL patient and healthy donor characteristics. [file 12967_2021_3136_MOESM2_ESM.docx]

**Table S1.** CLL patient and healthy donor characteristics.

| **Sample cohort** | **Number** | **Average age** | **Sex** |
| --- | --- | --- | --- |
| Healthy donor (in vitro study) | 6 | 70.5 +/- 6.3 (62-77) | 2 M /4 F |
| Healthy donor (ex vivo study) | 7 | 69.1 +/- 4.2 (64-77) | 4 M/ 3 F |
| Treatment naïve CLL | 11 | 66.2 +/- 8.7 (61-88) | 7 M /4 F |
| Ibrutinib | 7 | 63.1 +/- 11.2 (49-74) | 5 M/ 2 F |
| Zanubrutinib | 8 | 63.9 +/- 11.2 (46-79) | 6 M/ 2 F |
